# Host Plant Odors Represent Immiscible Information Entities - Blend Composition and Concentration Matter in Hawkmoths

**DOI:** 10.1371/journal.pone.0077135

**Published:** 2013-10-08

**Authors:** Anna Späthe, Andreas Reinecke, Alexander Haverkamp, Bill S. Hansson, Markus Knaden

**Affiliations:** Department of Evolutionary Neuroethology, Max Planck Institute for Chemical Ecology, Jena, Germany; INRA-UPMC, France

## Abstract

Host plant choice is of vital importance for egg laying herbivorous insects that do not exhibit brood care. Several aspects, including palatability, nutritional quality and predation risk, have been found to modulate host preference. Olfactory cues are thought to enable host location. However, experimental data on odor features that allow choosing among alternative hosts while still in flight are not available. It has previously been shown that *M. sexta* females prefer *Datura wrightii* compared to *Nicotiana attenuata*. The bouquet of the latter is more intense and contains compounds typically emitted by plants after feeding-damage to attract the herbivore’s enemies. In this wind tunnel study, we offered female gravid hawkmoths (*Manduca sexta*) odors from these two ecologically relevant, attractive, non-flowering host species. *M. sexta* females preferred surrogate leaves scented with vegetative odors form both host species to unscented control leaves. Given a choice between species, females preferred the odor bouquet emitted by *D. wrightii* to that of *N. attenuata*. Harmonizing, i.e. adjusting, volatile intensity to similar levels did not abolish but significantly weakened this preference. Superimposing, i.e. mixing, the highly attractive headspaces of both species, however, abolished discrimination between scented and non-scented surrogate leaves. Beyond ascertaining the role of blend composition in host plant choice, our results raise the following hypotheses. (i) The odor of a host species is perceived as a discrete odor ‘Gestalt’, and its core properties are lost upon mixing two attractive scents (ii). Stimulus intensity is a secondary feature affecting olfactory-based host choice (iii). Constitutively smelling like a plant that is attracting herbivore enemies may be part of a plant’s strategy to avoid herbivory where alternative hosts are available to the herbivore.

## Introduction

For insects that do not support their offspring after oviposition, the choice of host plant plays an essential role in ensuring reproductive success. To provide its offspring with an optimal environment, the parent needs to take into account several aspects, e.g. palatability, nutritional quality and shelter from enemies. The influence of these factors on host plant preference has been studied intensely [1-3], but the role of olfaction in mediating host choice is still unclear.

Here we investigate olfaction-guided host choice in gravid hawkmoth, *Manduca sexta*, females. Adult *M. sexta* forage on nectar from plants of several plant families, whereas oviposition occurs almost exclusively on solanaceous plants [4,5]. In the Great Basin Desert of Utah, *M. sexta* feed and oviposit on wild tobacco, *Nicotiana attenuata*, and jimsonweed, *Datura wrightii*; *Datura* is preferred both for nectar feeding [6] and oviposition [7]. Both species, in intact and non-flowering state, have been shown to emit overlapping but distinct volatile bouquets. Aside from qualitative differences, the less preferred *N. attenuata* is characterized by a total emission rate of volatile organic compounds that is 5 times greater than that of *D. wrightii* [7].

We ask (i) whether the preference for *D. wrightii* persists with the presentation of cues exclusively from the headspace. As the plant volatiles emitted by *D. wrightii* and *N. attenuata* differ both in quality and quantity [7], we also ask (ii) whether stimulus intensity, i.e. total volatile concentration, contributes to the differential attractiveness and (iii) whether the complete host blend, potentially perceived as an olfactory host image, is mediating host choice.

## Materials and Methods

### Insect and plants

*M. sexta* larvae were reared in laboratory as described in [8]. Pupae and adults were kept under an inverse 16 h : 8 h light/dark regime. Naïve females were mated the second night after emergence and tested during the subsequent night. Adults were supplied with sugar solution as desired. All plants were grown in a greenhouse as described [7]. Plants used for experiments were not yet flowering. Approximately 10 days before being used, plants were transferred into a climate chamber (23-25°C, 60-80% RH) with an inverse 16 h : 8 h light regime.

### Behavioral experiments

**Figure 1:**
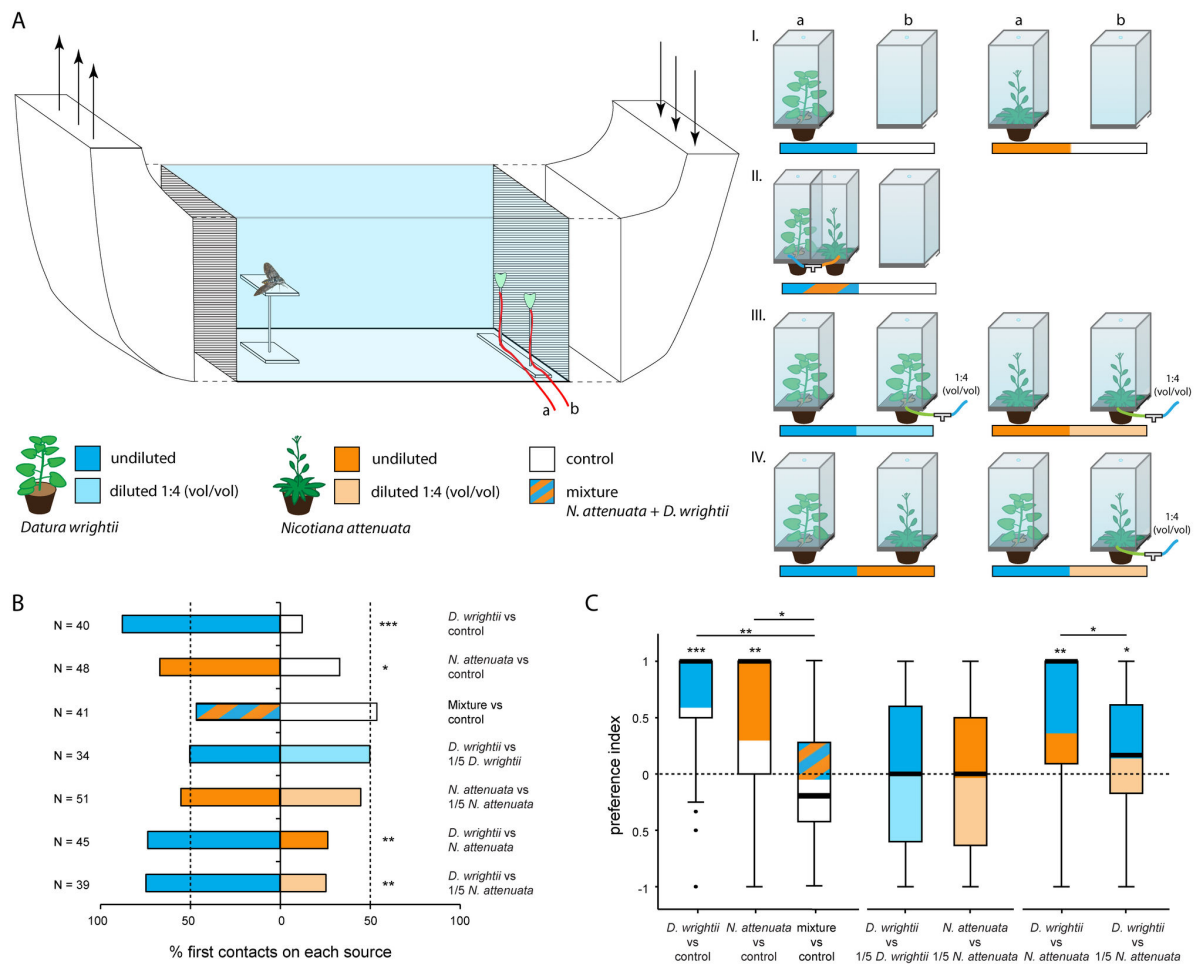
Effects of host blend composition and intensity on host choice in *M. sexta*. (A) Choice experiments with gravid *M. sexta* females were performed in a wind tunnel. Plants were placed in glass boxes outside the wind tunnel where they could not be seen by the moths. Pumps delivered plant headspace to two surrogate leaves serving as visual stimuli inside the wind tunnel. Two host plants, *D. wrightii* and *N. attenuata*, were tested (I) against a clean air control, (II) with their plant headspaces mixed together 1:1 against a clean air control, (III) against a conspecific plant whose headspace was diluted with clean air, and (IV) against each other, with *N. attenuata* headspace either not manipulated or diluted with clean air. Plant headspace and clean air were mixed in a 1:4 (vol/vol) ratio resulting in a 5-fold dilution. (B) The percentage of first choices made in the corresponding experiments. Sample size is given next to each experiment. Asterisks denote significant differences between sources (Binomial Test, *** p<0.001; ** p<0.01; *p<0.05). (C) Boxplots depict preference indices calculated from the number of contacts to each source. Values close to 1/-1 represent a high preference for one source; 0 means no preference. The black line delineates the median; color distribution within the box represents the percentage of contacts to each source. Asterisks above the boxes denote indices significantly different from 0 (Wilcoxon Signed Ranks Test, *** p<0.001; ** p<0.01; * p<0.05). Preference indices resulting from experiments in which the plant headspace of both species is offered superimposed or separately against clean air differed significantly (Kruskal-Wallis Test, p<0.0001, and Dunn’s post hoc test, ** p<0.01, * p<0.05). Furthermore, preference indices derived from interspecific choice experiments were significantly different from each other (Mann-Whitney U Test, p<0.05).

The preference of gravid moths for plant headspace, including odors, CO_2_ and humidity, emitted by the host species *D. wrightii* and *N. attenuata* was assessed in wind tunnel experiments (Figure 1A). Headspace from each species was compared individually and in a mixture against a clean air control (Figure 1A-I, II). The effects of signal intensity were tested within (Figure 1A-III) and between both species (Figure 1A-IV); see ‘stimulus delivery’ below.

### Wind tunnel assay

The wind tunnel (Plexiglas, L x H x W 2.5 x 1 x 1 m) was set to an airflow of 0.4ms^-1^, 25°C, and 70% RH. LED stripes (peak wavelength white: 448 nm, infrared: 846 nm) fixed at an angle to the side walls of the wind tunnel chamber provided continuous, indirect illumination at 0.3 Lux. Mated females were tested individually during the first 3 h of scotophase. 1 h before the experiment, females were transferred individually into mesh tubes (13 x 15cm). These served to place the females without further handling at the start of each experiment on a release platform. Females that did not show wing-fanning behavior within 3 min were gently prodded. Unlike no-choice wind tunnel experiments, where time until activation can be a critical parameter, activating the females does not bias the results in choice-based experiments. We observed the females for 4 min after take-off, noting every event involving physical contact with legs or ovipositor to the surrogate leaves at the odor sources. Since the choice between scented leaf dummies was the experimental criterion, only females that contacted the source were evaluated. Females that did not show wing-fanning behavior within 5 min or did not contact the odor sources were regarded as non-responders and excluded from the statistical analysis (see Table 1). Females showing proboscis extension, i.e. nectar-foraging, were also excluded. We evaluated first choices and preference indices based on the total number of repeated contacts to the sources: ((contacts A – contacts B) / total contacts) ranging from 1 (absolute preference of source A) to -1 (absolute preference of source B).

**Table 1:** Numbers of responders, non-responders and animals excluded from analysis due to their behaviour of extending their proboscis during the experiment are given for each experiment.

		**excluded**	**non-responders**		**responders**			
**Experiment**	**total**	**Proboscis**	**no take off**	**no contact**	**contact**	**number of contacts (± SD)**		**Significance** ^a.^
*D. wrightii*						*D. wrightii*	2.9 ± 2.9	**dF=38**
vs	83	3	1	39	40			
control						control	0.7 ± 1.1	**p < 0.001**
*N. attenuata*						*N. attenuata*	2.1 ± 2.4	**dF=43**
vs	122	6	0	68	48			
control						control	0.8 ± 1.4	**p < 0.001**
mixture						mixture	3.0 ± 2.4	dF=37
vs	77	10	19	7	41			
control						control	3.9 ± 2.9	n.s.
diluted *N. attenuata*						1/5 N*. Attenuata*	2.5 ± 2.1	dF=46
vs	72	5	5	11	51			
*N. attenuata*						*N. attenuata*	2.4 ± 2.6	n.s
diluted *D. wrightii*						1/5 D*. wrightii*	2.2 ± 2	dF=30
vs	56	4	9	9	34			
*D. wrightii*						*D. wrightii*	2.6 ± 2.8	n.s.
*N. attenuata*						*N. attenuata*	1.1 ± 1.5	**dF=44**
vs	81	5	0	31	45			
*D. wrightii*						*D. wrightii*	2.5 ± 3	**p < 0.01**
diluted *N. attenuata*						1/5 N*. Attenuata*	2.7 ± 2.3	**dF=33**
vs	55	3	10	3	39			
*D. wrightii*						*D. wrightii*	4 ± 3.3	**p < 0.05**

Average number of source contacts (± SD) was tested with Wilcoxon Signed Rank Test.

a Wilcoxon Signed Rank Test

### Stimulus delivery

All experiments were performed with plant headspace volatiles produced in real time delivered from two sources 40 cm apart at the upwind end of the tunnel (Figure 1A). 1 h before the experiment, plants were placed in glass boxes (L x W x H: 40 x 40 x 60 cm; glass panes fixed in aluminium frames) outside the wind tunnel. Two aluminum panes had V-shaped, guillotine-like front ends that fit into the notches of the lower frames of the glass box. Gently adjusted around the stems of experimental plants, they served to exclude rhizosphere volatiles from the headspace of the shoot. Active charcoal-filtered air (1.2 L/min) was introduced through a diffuser at the top of the glass boxes. Volatile-laden headspace air was removed close to the stem at the base of the box, pumped into the wind tunnel (0.8 L/min) and released continuously during the experiment below an artificial leaf (approximately 6 x 8 cm) made of light green tissue paper (Figure S1).

The total volatile emission of *N. attenuata* was found to be five times higher than that of *D. wrightii* [7]. To investigate the importance of stimulus intensity, i.e. plant headspace concentrations for ovipositing hawkmoths, AC-filtered air was added at a 1:4 (vol/vol) ratio via a y-connector. The efficient dilution of a reference headspace applying the experimental methods as described has been shown in a series of control experiments; see supplement. We asked whether (i) a diluted stimulus would be as attractive as the original plant headspace and (ii) a volatile concentration of a *N. attenuata* headspace adjusted to *D. wrightii* concentrations would reveal concentration effects in host species preferences.

To investigate whether the preference for *D. wrightii* might be linked to an olfactory host image that reflects the volatiles emitted by the plant, we superimposed the headspace of *N. attenuata* on that of *D. wrightii*, mixing them 1:1 (vol/vol) via a y-connector, and tested this mixture against clean air.

## Results

### Plants versus controls

For their first contact, females significantly preferred the headspaces of *D. wrightii* and *N. attenuata* over those of clean air controls (Figure 1B; *D. wrightii*: p<0.001; *N. attenuata*: p<0.05; Binomial Test). The total number of contacts with plant odor sources was significantly higher compared to the number of contacts with the control source (Table 1), resulting in a significant preference index (Figure 1C). When the two host blends were mixed, any preference for plant headspace was completely abolished (Figure 1 B, C; Table 1). Consequently, the preference index of the superimposed host blend experiment differed significantly from the indices of the experiments testing the species singly against clean air (Figure 1C; p<0.0001; Kruskal-Wallis Test; dF=2).

### Signal attenuation: intraspecific

No preference was observed when headspace from *N. attenuata* or *D. wrightii* was presented against diluted headspace from the same species for both first contacts (Figure 1B) or for the total number of contacts (Figure 1C; Table 1; p>0.05; Wilcoxon Signed Ranks Test).

### Signal attenuation: interspecific

Comparing non-manipulated plant headspaces, females showed significantly more first contacts (Figure 1B) and a significantly higher total number of contacts (Figure 1C; Table 1; p<0.01; Wilcoxon Signed Ranks Test; dF=44) to *D. wrightii*-scented sources than to *N. attenuata*-scented sources. Diluting the *N. attenuata* headspace in a ratio of 1:4 (vol/vol) with clean air to a volatile concentration similar to that of *D. wrightii* did not abolish the previously observed bias of first contacts and total number of contacts towards *D. wrightii* (Figure 1C; Table 1). Nevertheless, comparing preference indices between the experiments revealed that diluting the *N. attenuata* blend significantly weakened the preference for *D. wrightii* (Figure 1C; p<0.05; Mann-Whitney U Test).

## Discussion

We show that volatile airborne stimuli alone are sufficient to elicit a differential preference of host plant species in mated *M. sexta* females. Headspace compounds from both species are attractive when compared to clean air, and the attractiveness is robust against a reduction in stimulus intensity. When spatially separated sources of *N. attenuata* and *D. wrightii* headspaces were presented simultaneously, insects showed a strong preference for the latter. This olfaction-mediated host choice corresponds to oviposition preferences previously demonstrated in *Manduca* [7].

*N. attenuata* plants emit a 5-fold higher amount of volatiles compared to *D. wrightii* [7]. Although *D. wrightii* headspace was still significantly preferred when volatile concentrations in the *N. attenuata* headspace were reduced to the level of the former, the comparison of preference indices revealed a significant attenuation of the host preference. Thus, stimulus intensity modifies olfactory host preference at the species level in a repeated choice setting.

The observed hierarchy of preference, which is consistent with what has previously been reported [7], underlines the prominent role played by *D. wrightii* as a host plant for *M. sexta*. The attractiveness of this species has been associated with the presence of large, night-blooming and intensely scented flowers that provide large amounts of nectar to the moth [6,9]. However, to uncouple host from foraging cues, we presented volatiles from non-flowering plants and excluded from analysis those animals that extend their proboscis during flight. Our results demonstrate that, as flower-derived odors do [10,11], vegetative plant volatiles play an important role in mediating host choice.

Carbon dioxide (CO_2_) emissions serve *M. sexta* to assess nectar abundance in flowers [12,13]. CO_2_ is also a close-range oviposition attractant in other insects, e.g. [14,15]. Plant respiration during the scotophase results in CO_2_ emissions and *M. sexta* sense fluctuations in CO_2_ concentration [16]. It is therefore conceivable that this gas and humidity serve to attract *M. sexta* females to host plants or help the moths to assess plant vigor. Preliminary data gathered in a different context indicate that individual *D. wrightii* and *N. attenuata* plants as used in this experiment emit CO_2_ at 39.7 ± 30.40 (SD) and 26.5 ± 21.02 (SD) ppm above ambient levels, and relative humidity 9.8 ± 3.79 (SD) and 6.5 ± 1.32 (SD) percent above ambient levels, respectively. In our experiments, diluted plant headspace was as attractive as the non-diluted conspecific headspace. Mixing *N. attenuata* and *D. wrightii* headspaces abolished the preference female *M. sexta* had shown for each individual headspace when compared to clean air. We cannot exclude that CO_2_ and humidity may co-attract insects or help them assess plant vigor when choosing among different plants. But neither of them overrode the combined effect of all natural plant headspace compounds.

Insects may extract information about a volatile-emitting plant through characteristic compounds or compound classes [17-19], and species-specific plant volatile blends [17,20]. Stimulus concentration has to our knowledge not been addressed as part of the mechanism that codes host information. A comparison of the volatile profiles of *N. attenuata* and *D. wrightii* revealed qualitative as well as quantitative differences: in undamaged plants, the volatile emissions of *N. attenuata* were 5 times higher than those of *D. wrightii* (total emission rate of *D. wrightii*: 1.8 ± 0.3 ng/min; total emission rate of *N. attenuata*: 10.5 ± 0.5ng/min [7]), and *N. attenuata* headspace was composed of many typical herbivore-induced plant volatiles (HIPVs), i.e. compounds whose emissions are up-regulated after herbivore attack [21]. It has been shown that HIPVs mediate avoidance of damaged plants in ovipositing moths [22,23]. Thus, the preference for *D. wrightii* compared to *N. attenuata* may be co-mediated by volatiles associated with herbivore-damaged plants. Consequently, reduced HIPV-concentrations might lead to the shifted preference indices observed in our experiments, and conversely enhanced HIPV emissions may protect the emitter as long as the herbivore may choose among alternative hosts in a field setting.

When we presented moths with different concentrations of two blends of identical compositions, they did not discriminate between full and diluted plant headspaces. In a turbulent environment distance from a stimulus source has been shown to be mainly coded by stimulus intermittency [24], differences in plant volatile concentration could also have been interpreted by the moths as a function of distance to the plant [25]. However, our results give no indication that a moth would prefer a host-plant blend at a higher concentration. Hence, ovipositing moths seem to rely predominantly on blend composition rather than on concentration.

The picture changed when we diluted the *N. attenuata* blend, bringing its total concentration to the level of *D. wrightii*, and presented both simultaneously. While *D. wrightii* is usually strongly preferred over *N. attenuata*, this preference became less accentuated when the odor of *N. attenuata* was diluted (Figure 1C). Thus, stimulus intensity may become a supplemental behaviorally relevant feature of an odor stimulus when different, i.e. species specific odor blends are available in a repeated choice setting, which is the standard case in the field. In support of these lines, *M. sexta* females preferred to oviposit on inbred horse nettle plants, which emit considerably fewer volatiles compared to outbred plants [26].

The preference shown for single plant blends rather than clean air disappeared when *N. attenuata* and *D. wrightii* blends were presented as a mixed stimulus. Clearly, *M. sexta* females could no longer evaluate the olfactory information provided by this combined species mixture, suggesting that the composition of the species-specific blend contains crucial information, an olfactory ‘Gestalt’. In Colorado beetles, adding single host volatiles [27] or a non-host plant [28] to the blend of a host plant has been shown to neutralize the host’s attractiveness. Host recognition in *M. sexta* females is very likely also dependent on ratio specificity. Several studies have reported that the emissions of host plants can be masked by repellent or neutral blends [29]. However, the reciprocal neutralization of two attractive blends from two naturally preferred host plants shown in this study has to our knowledge never been reported.

We showed that for a *Manduca sexta* female, the olfactory information emitted by host plants is sufficient to mediate its choice among alternative hosts. Furthermore, our results highlight that species-specific host odours represent information entities that loose their positive valence upon blending, and that stimulus intensity is a supplemental feature involved in choosing among alternative hosts. Constitutively emitting HIPV at a higher rate contributes to an herbivores preference for the alternative host and may, thus, be advantageous to the smelly plant.

## Supporting Information

Figure S1 **Experimental manipulation of plant headspace.** (A) Experimental setup to generate headspace dilution and mixtures. Plants were placed in glass boxes (GB) and provided with active charcoal (AC) filtered air from the top. Two metal plates (MP) with a central opening that were located on metal slides beneath the box enclosed the plant stem close to the pot, thereby excluding roots and soil material from the box. Rotary vane vacuum and pressure pumps (RP; G12/01 EB, Gardner Denver, Inc., Puchheim, Germany) delivered plant headspace out of the glass box, and the resulting headspace flow was controlled with mechanical valves. To establish plant headspace of 1:4 dilutions and 1:1 mixtures, both flows were adjusted to the aimed flow ratio and merged in a Y-shaped connector. During the experiment, flow rates of the source flows (S1, S2) as well as the total flow (TF) were repeatedly checked with a digital flowmeter. (B) Differences in acetone concentration resulting from the flow ratios used during the experiments were measured with a fast response Photo-Ionization detector (PID) (miniPID 200A, Aurora Scientific Inc., Ontario, Canada) for a time window of 60 s. The internal offset and gain of the PID had been set to 0 and 1, respectively. Measurements were transferred to a personal computer (Pentium 4, 2.8GHz, Fujisu Siemens, Munich, Germany) via an analog-digital converter (National Instruments, Austin, Texas, US). Data acquisition within the entire system was adjusted to the PID’s maximum sampling rate of approximately 330Hz. During the test a 50% aqueous acetone solution was placed in the glass box to simulate plant headspace. Acetone headspace was pumped out of the box and regulated with valves to simulate non-manipulated (dark green), 1:1 mixed (green) and 1:4 diluted (light green) headspace. Clean air pumped out of a glass box served as a control measurement (blue line) and a second source for the 1:1 mixture. 1:4 dilutions were established directly with AC-filtered air. Non-manipulated headspace was measured before (solid line) and after (dotted line), adjusting the valves to ensure that the acetone concentration in the glass box did not drop. (C) Boxplots show the average acetone concentration in V resulting from PID measurements. By adjusting the flow ratio the non-manipulated acetone concentration (100%) reached the hoped-for concentration levels of 50 and 20% (dotted lines), representing the 1:1 mixture and 1:4 dilution, respectively.
(TIF)
