# Supplementary figures and images for: Host Plant Odors Represent Immiscible Information Entities - Blend Composition and Concentration Matter in Hawkmoths
Source: PLoS One. 2013 Oct 8;8(10):e77135. doi: 10.1371/journal.pone.0077135 (PMC3792911; doi:10.1371/journal.pone.0077135)

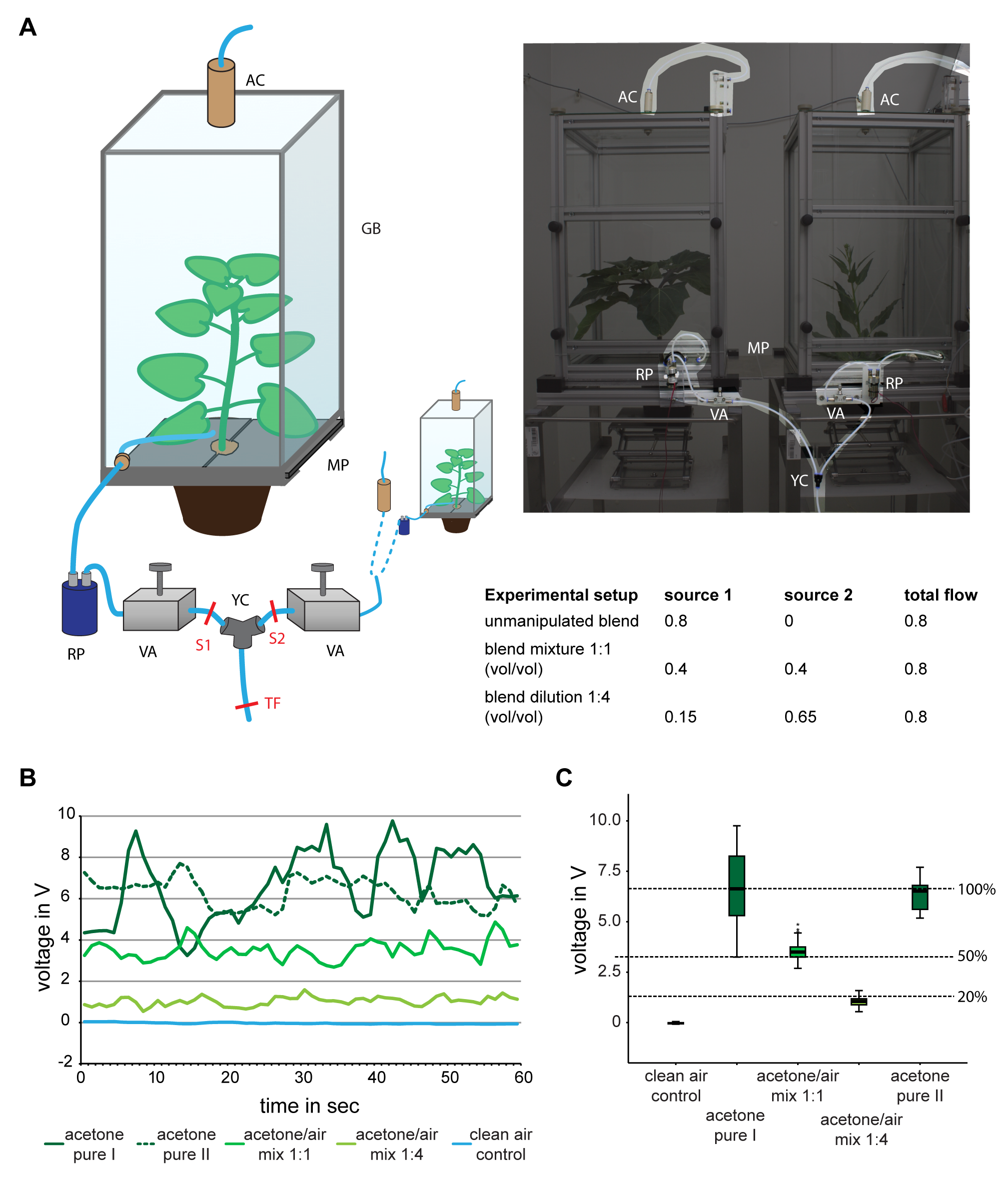

Supplement: Figure S1 — Experimental manipulation of plant headspace. (A) Experimental setup to generate headspace dilution and mixtures. Plants were placed in glass boxes (GB) and provided with active charcoal (AC) filtered air from the top. Two metal plates (MP) with a central opening that were located on metal slides beneath the box enclosed the plant stem close to the pot, thereby excluding roots and soil material from the box. Rotary vane vacuum and pressure pumps (RP; G12/01 EB, Gardner Denver, Inc., Puchheim, Germany) delivered plant headspace out of the glass box, and the resulting headspace flow was controlled with mechanical valves. To establish plant headspace of 1:4 dilutions and 1:1 mixtures, both flows were adjusted to the aimed flow ratio and merged in a Y-shaped connector. During the experiment, flow rates of the source flows (S1, S2) as well as the total flow (TF) were repeatedly checked with a digital flowmeter. (B) Differences in acetone concentration resulting from the flow ratios used during the experiments were measured with a fast response Photo-Ionization detector (PID) (miniPID 200A, Aurora Scientific Inc., Ontario, Canada) for a time window of 60 s. The internal offset and gain of the PID had been set to 0 and 1, respectively. Measurements were transferred to a personal computer (Pentium 4, 2.8GHz, Fujisu Siemens, Munich, Germany) via an analog-digital converter (National Instruments, Austin, Texas, US). Data acquisition within the entire system was adjusted to the PID’s maximum sampling rate of approximately 330Hz. During the test a 50% aqueous acetone solution was placed in the glass box to simulate plant headspace. Acetone headspace was pumped out of the box and regulated with valves to simulate non-manipulated (dark green), 1:1 mixed (green) and 1:4 diluted (light green) headspace. Clean air pumped out of a glass box served as a control measurement (blue line) and a second source for the 1:1 mixture. 1:4 dilutions were established directly with AC-filtered air [file pone.0077135.s001.tif]
